# A Rare Case of Garre’s Osteomyelitis of Tibia in an Adult

**DOI:** 10.7759/cureus.54034

**Published:** 2024-02-11

**Authors:** Kushagra Kaushik, Gopal T Pundkare, Anish Tawde, Kannan A

**Affiliations:** 1 Department of Orthopaedics, Bharati Vidyapeeth Deemed University Medical College, Pune, IND; 2 Department of Arthroplasty, KIMS-Sunshine Hospital, Hyderabad, IND

**Keywords:** surgical debridement, mri, tibial lesion, proliferative periostitis, chronic osteomyelitis, garre's osteomyelitis

## Abstract

To explore a rare case of Garre's osteomyelitis in an adult, typically observed in children, and detail its diagnostic and treatment approach, we conducted a case study of a 40-year-old male presenting with persistent right tibial pain.

Through diagnostic procedures, including radiography and MRI, a broad differential diagnosis was established. Histopathological examination post-surgical intervention confirmed Garre's osteomyelitis. The treatment, which included corticotomy debridement, saucerization, ceramic granules insertion, and targeted antibiotic therapy, resulted in significant improvement over one year.

This case underscores the importance of considering Garre's osteomyelitis in the differential diagnoses of chronic tibial lesions in adults and highlights the necessity of a comprehensive diagnostic and treatment approach in managing such rare cases, thus contributing valuable insights to orthopedic practice and literature.

## Introduction

Garre's osteomyelitis, first identified by Carl Garre in 1893, is a unique chronic osteomyelitis characterized by new bone formation and a thickened periosteum without pus [1]. Predominantly seen in children, its pathogenesis often follows minor trauma or infection, yet it remains poorly understood. Rare in adults, it presents significant diagnostic challenges due to its atypical symptoms and resemblance to other bone diseases [2].

Clinically, patients exhibit localized pain and swelling, often without systemic symptoms like fever, leading to a delayed diagnosis [3]. Radiologically, it presents with distinctive new bone layers, which can be subtle and mistaken for other conditions, such as neoplastic lesions [4]. The rarity in adults demands careful differentiation from a wider range of diseases, including bone tumors.

Treatment involves surgical debridement and antibiotics, with *Staphylococcus aureus* as the common pathogen [5]. This report focuses on an adult case of Garre's osteomyelitis, highlighting the need for its consideration in differential diagnosis and discussing its clinical, radiological, and treatment nuances.

## Case presentation

A 40-year-old female with no significant past medical history presented to the orthopedic clinic with a six-month history of persistent, dull, and aching pain in the right tibia. The patient described the pain as non-radiating and localized, with a gradual onset and progressive nature. Notably, she denied any recent trauma, fever, weight loss, or other systemic symptoms. The patient was a homemaker, which involved regular physical activity, and she reported that the pain exacerbated with exertion and was somewhat relieved with rest.

**Figure 1:**
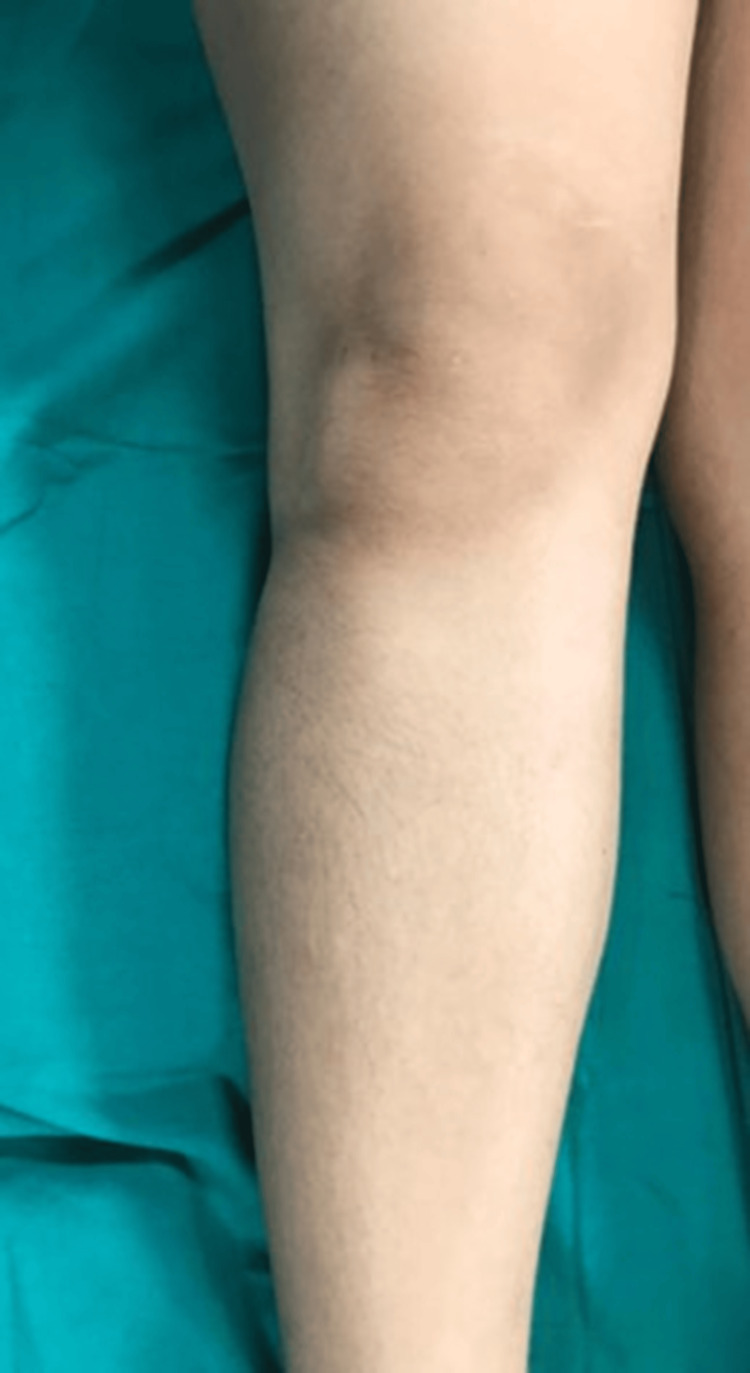
On physical examination, there was no apparent deformity or overlying skin changes

On physical examination, there was no apparent deformity or overlying skin changes (Figure 1).

However, palpation revealed diffuse tenderness over the proximal third of the right tibia, and a firm, immovable mass was felt deep into the soft tissues. There was no increased warmth, erythema, or regional lymphadenopathy. The range of motion in the adjacent knee and ankle joints was unaffected.

**Figure 2:**
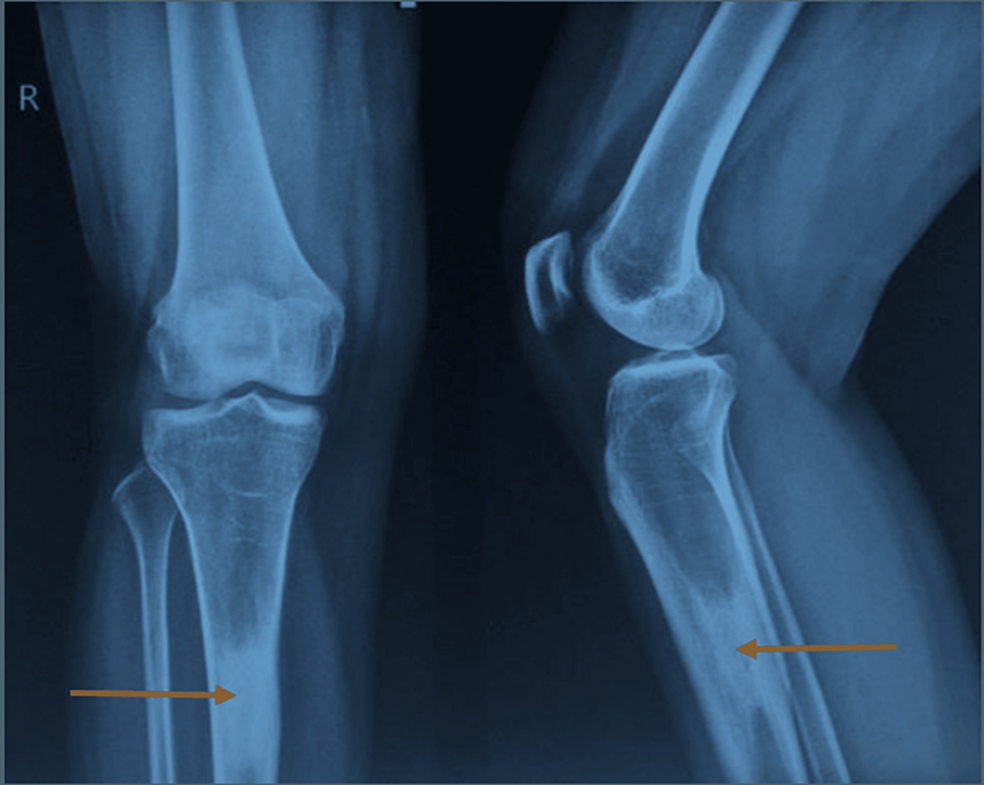
Right leg X-ray images (AP and lateral projections) showed an ill-defined sclerotic lesion in the proximal diaphysis of the tibia with a small, round lytic area

Radiographs of the right tibia showed an ill-defined sclerotic lesion in the proximal diaphysis with a small round lytic area suggestive of a chronic process (Figure 2).

**Figure 3:**
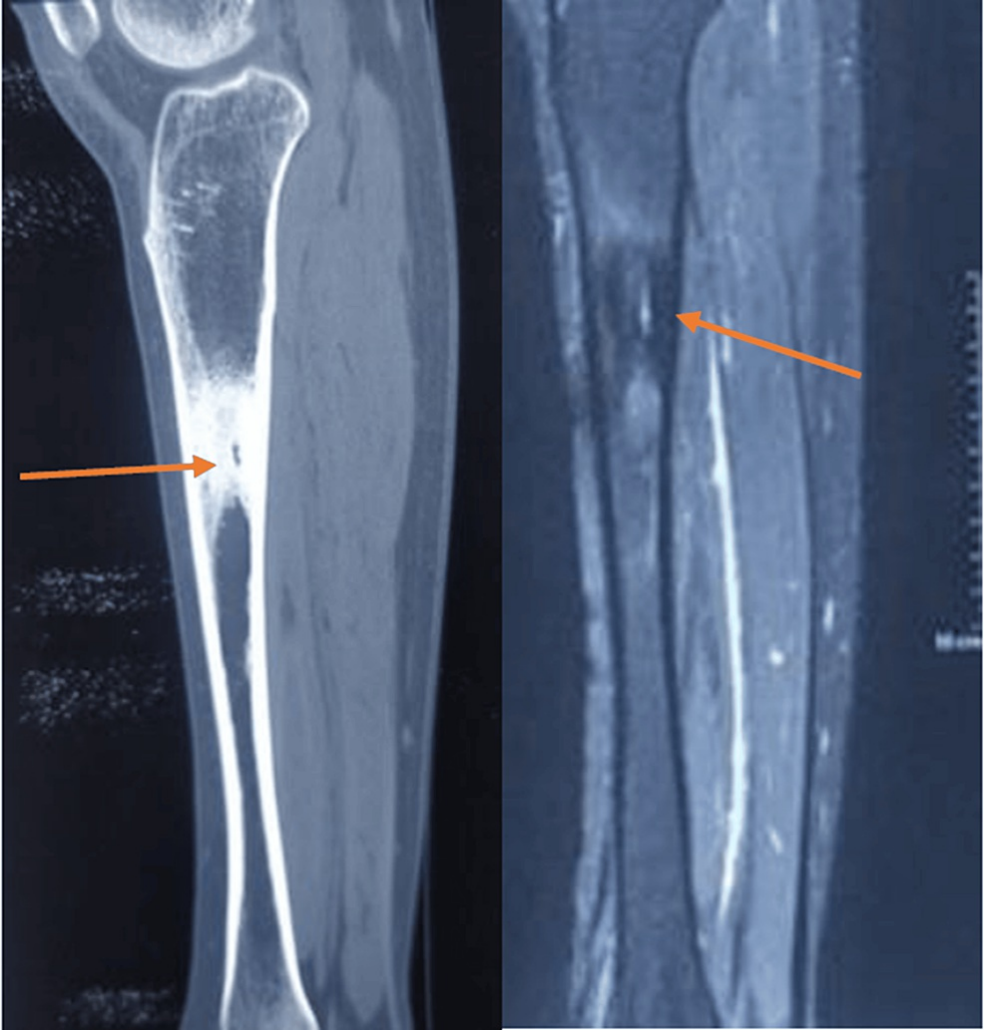
Sagittal MRI T1 and T2 sequences revealed thickening of the periosteum of the tibia and a well-defined area of cortical disruption

Given the patient's age and occupation, initial differential diagnoses included stress fracture, chronic osteomyelitis, osteoid osteoma, and bone neoplasms. To further elucidate the nature of the lesion, a magnetic resonance imaging (MRI) scan was performed, which revealed thickening of the periosteum and a well-defined area of cortical disruption consistent with chronic inflammatory changes rather than a neoplastic process (Figure 3).

**Figure 4:**
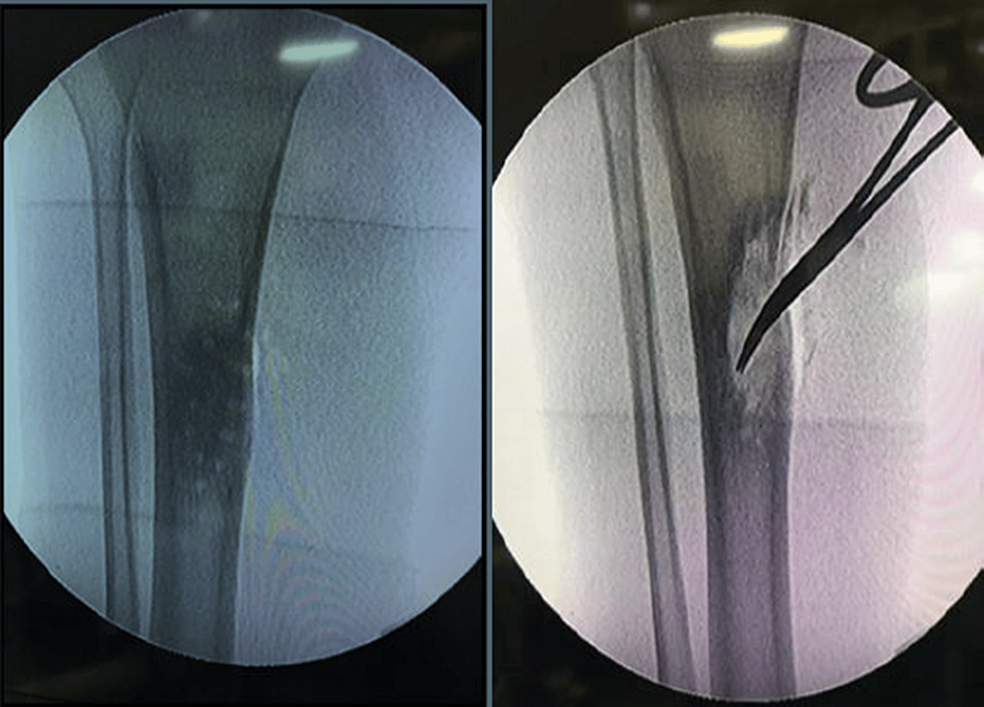
Patient underwent a corticotomy debridement with saucerization and ceramic granules insertion

The patient underwent a corticotomy debridement with saucerization and antibiotic-mixed ceramic granule insertion under general anesthesia (Figure 4).

**Figure 5:**
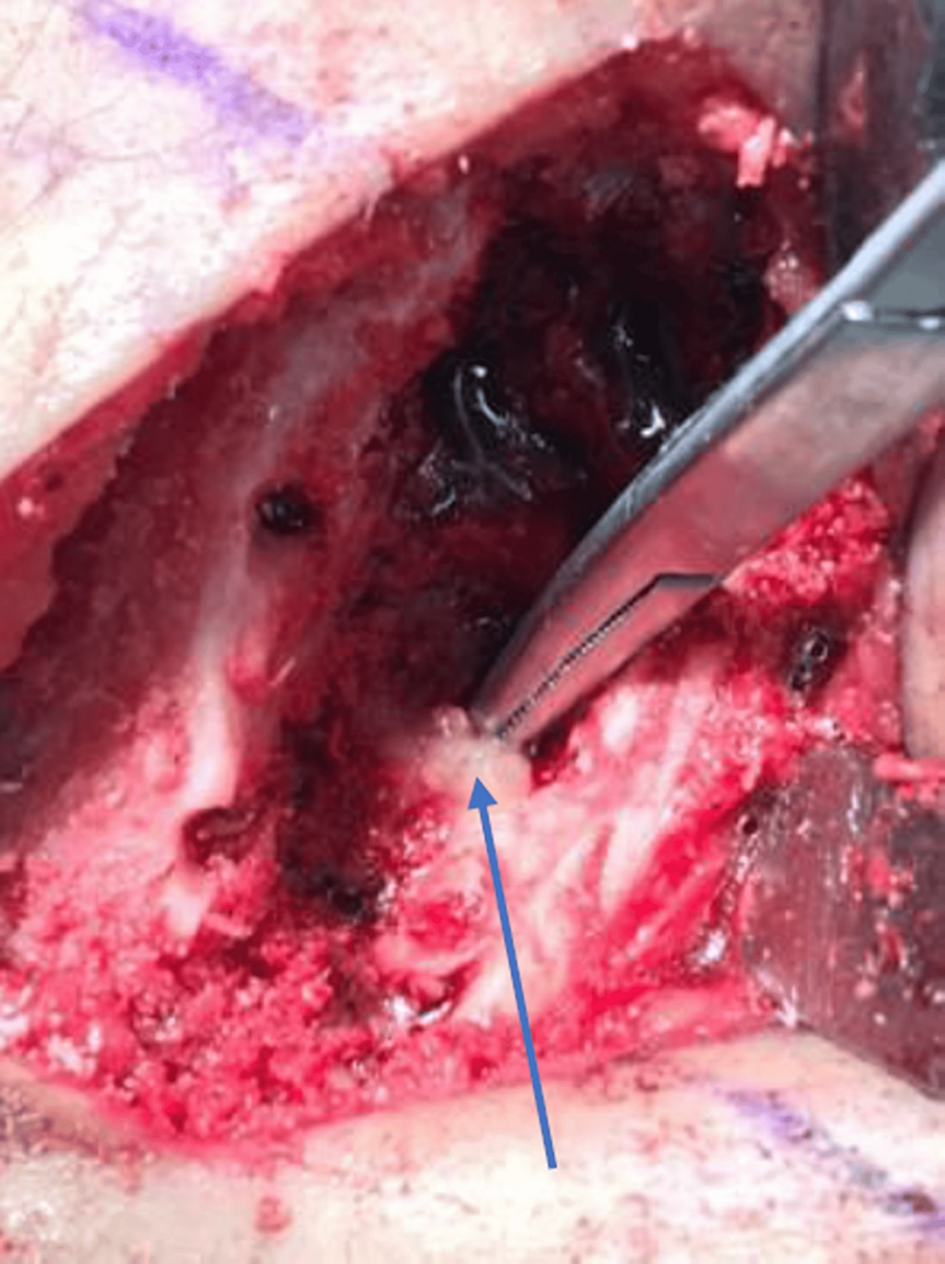
Intraoperatively, a dense sclerotic bone with an overlying thickened periosteum containing some purulent material was noted

Intraoperatively, a dense sclerotic bone with an overlying thickened periosteum was noted (Figure 5).

Some purulent material was encountered, and samples were taken for culture and histopathology. The postoperative period was uneventful, and the patient was started on empirical antibiotic therapy.

**Figure 6:**
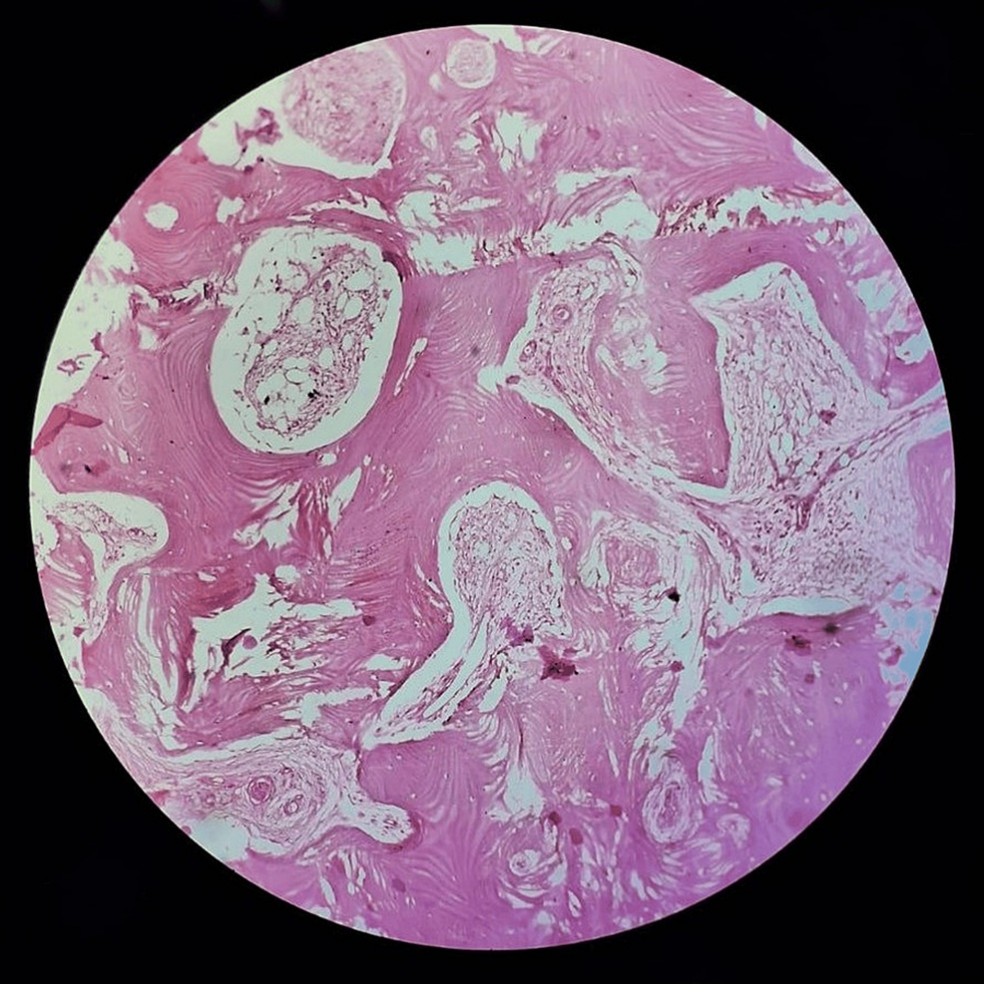
Histopathological examination showed bony trabeculae surrounded by dense fibrous and fibrovascular tissue with areas of chronic inflammatory cells

Culture results later identified *Staphylococcus aureus*, and antibiotic therapy was adjusted accordingly. Histopathological examination showed bony trabeculae surrounded by dense fibrous and fibrovascular tissue with areas of chronic inflammatory cells, confirming the diagnosis of Garre’s osteomyelitis (Figure 6).

The patient was followed up regularly in the outpatient department. At the three-month follow-up, she reported significant pain relief and had resumed her normal activities. Follow-up radiographs showed evidence of healing with recalcification of the lytic areas. At the one-year mark, the patient remained symptom-free with no signs of recurrence.

## Discussion

This case presents a rare instance of Garre’s osteomyelitis in an adult patient, a condition more typically seen in the pediatric population. The diagnosis of Garre's osteomyelitis in adults is challenging due to its rarity and the subtlety of its presentation, as highlighted in our patient. The absence of systemic inflammatory signs, such as fever or leukocytosis, further complicates the clinical picture, making it easy to overlook in the initial differential diagnosis [1].

Garre's osteomyelitis is characterized by a sclerosing form of chronic osteomyelitis with new bone formation and a thickened periosteum. In the current case, the radiographic findings of an ill-defined sclerotic lesion with a lytic area were initially perplexing. Such imaging findings are nonspecific and can mimic a variety of bone pathologies, including neoplastic processes. This diagnostic dilemma necessitates consideration of Garre's osteomyelitis in the differential diagnosis of sclerotic bone lesions, particularly when the clinical presentation is atypical, as in this case [2].

The decision to proceed with surgical intervention in the present case was based on the persistent pain and the need to obtain tissue for a definitive diagnosis. The surgical findings of dense sclerotic bone and a thickened periosteum were consistent with Garre's osteomyelitis. The absence of purulent material further supported this diagnosis over a more acute infectious process.

Histopathological examination is crucial in confirming the diagnosis of Garre’s osteomyelitis. In this case, the presence of dense fibrous and fibrovascular tissue with chronic inflammatory cells was consistent with the disease. These findings were instrumental in differentiating it from other conditions, such as osteoid osteoma or bone malignancies, which can present similarly [3].

The successful management of Garre’s osteomyelitis requires a combination of surgical and medical approaches. The surgical debridement helps in removing the sequestrum and reducing the bacterial load, while antibiotic therapy addresses the underlying infection. In this case, the identification of *Staphylococcus aureus* and the subsequent targeted antibiotic therapy were pivotal in the patient's recovery.

This case also highlights the importance of long-term follow-up in managing chronic osteomyelitis. The recurrence of Garre’s osteomyelitis is a possibility, particularly if the initial management is inadequate. Regular follow-up ensures early detection and intervention in cases of recurrence [5].

In conclusion, Garre’s osteomyelitis should be considered in adult patients presenting with chronic, localized bone pain and radiographic evidence of sclerotic lesions. A multidisciplinary approach involving clinical evaluation, imaging, histopathology, and appropriate surgical and medical management is key to successful outcomes in such cases.

## Conclusions

In reflecting on this case, it is evident that Garre's osteomyelitis, though rare, should maintain a place in the differential diagnosis for adult patients presenting with chronic bone lesions. Early recognition and appropriate management are paramount to achieving favorable outcomes and preventing long-term complications. Furthermore, this case contributes to the growing body of literature on the condition and serves as an educational cornerstone for clinicians, enhancing understanding and fostering improved patient care.

Ultimately, this case exemplifies the intricate interplay between patient presentation, clinical suspicion, and the judicious use of diagnostic and therapeutic modalities in orthopedic practice. It reinforces the notion that a rare diagnosis should not be overlooked, even in populations where it is uncommon. By sharing these insights, the present case report aims to enrich the clinical acumen of healthcare professionals and improve outcomes for patients with similar rare and challenging conditions.
